# May the 4C's be with you: an overview of complexity-inspired frameworks for analysing resting-state neuroimaging data

**DOI:** 10.1098/rsif.2022.0214

**Published:** 2022-06-29

**Authors:** Fran Hancock, Fernando E. Rosas, Pedro A. M. Mediano, Andrea I. Luppi, Joana Cabral, Ottavia Dipasquale, Federico E. Turkheimer

**Affiliations:** ^1^ Department of Neuroimaging, Institute of Psychiatry, Psychology and Neuroscience, King's College London, London, UK; ^2^ Centre for Psychedelic Research, Department of Brain Science, Imperial College London, London SW7 2DD, UK; ^3^ Data Science Institute, Imperial College London, London SW7 2AZ, UK; ^4^ Centre for Complexity Science, Imperial College London, London SW7 2AZ, UK; ^5^ Department of Psychology, University of Cambridge, Cambridge CB2 3EB, UK; ^6^ Department of Psychology, Queen Mary University of London, London E1 4NS, UK; ^7^ Division of Anaesthesia, School of Clinical Medicine, University of Cambridge, Cambridge, UK; ^8^ Department of Clinical Neurosciences, University of Cambridge, Cambridge, UK; ^9^ Leverhulme Centre for the Future of Intelligence, University of Cambridge, Cambridge, UK; ^10^ Alan Turing Institute, London, UK; ^11^ Life and Health Sciences Research Institute (ICVS), School of Medicine, University of Minho, Braga, Portugal; ^12^ Department of Psychiatry, University of Oxford, Oxford, UK

**Keywords:** complexity, connectivity, computation, criticality, metastability, integrated information

## Abstract

Competing and complementary models of resting-state brain dynamics contribute to our phenomenological and mechanistic understanding of whole-brain coordination and communication, and provide potential evidence for differential brain functioning associated with normal and pathological behaviour. These neuroscientific theories stem from the perspectives of physics, engineering, mathematics and psychology and create a complicated landscape of domain-specific terminology and meaning, which, when used outside of that domain, may lead to incorrect assumptions and conclusions within the neuroscience community. Here, we review and clarify the key concepts of connectivity, computation, criticality and coherence—the 4C's—and outline a potential role for metastability as a common denominator across these propositions. We analyse and synthesize whole-brain neuroimaging research, examined through functional magnetic imaging, to demonstrate that complexity science offers a principled and integrated approach to describe, and potentially understand, macroscale spontaneous brain functioning.

## 1. Introduction

The orchestrated activity of the approximately 100 billion neurons connected via an estimated 200 trillion synapses in the human brain [1] is certainly perplexing, and its explanation has allured scientists for more than 100 years [2]. A popular way to approach this challenge has been to see the brain as a computer, i.e. a physical instantiation of algorithms that works on inputs from various sensory systems in order to generate behaviour [3–6]. The computational view of the brain has introduced important new conceptual resources to neuroscience, providing a (in principle) feasible roadmap of how one could attempt to understand the brain—e.g. through Marr's celebrated ‘three levels of analysis' [7].

While the brain can be seen as a computer, it is first and foremost a living organ driven by metabolic and thermodynamic constraints. In effect, while an uninformed computational view could suggest that the brain is ‘idle’ when not actively engaging with a specific task, early evidence has shown that the brain accounts for 20% of the body's energy consumption while making up just 2% of body weight [8]. In effect, brain functional activity may be spontaneous or evoked with cognitive, behavioural or motor tasks, and the metabolic cost of spontaneous or intrinsic activity far exceeds the costs for evoked activity [9]. Despite this evidence, and the work of notable pioneers such as Freeman [10], neuroimaging data from resting-state conditions did not attract as much attention within the neuroimaging community as data from task-related conditions. Nonetheless, there is nowadays a growing consensus that making sense of spontaneous brain activity is crucial for understanding brain function [11–13].

Among the available neuroimaging techniques, functional magnetic resonance imaging (fMRI) provides a modality to probe resting-state whole-brain activity at a high spatial resolution. Evidence has repeatedly shown that the resulting time series of resting-state experiments are highly structured, in a way that is not easy to explain purely in terms of an input/output information-processing perspective. Here, we argue that complexity science provides an important complement to the computational view for building explanations on the nature of these data.

Complexity science aims to identify common laws that govern complex systems made by multiple interactive elements, bringing together tools from statistical physics, dynamical systems theory (DST), information theory (IT) and other fields [14–16]. Although this multiplicity of approaches brings richness to the research, it can also inadvertently lead to inconsistent use of terminology, misunderstandings, and to potentially inconsistent conclusions. Adding to these problems, neuroscientists face the challenge of translating diverse domain-specific conceptual theories and models into plausible biophysical mechanisms. Acknowledging the magnitude of this challenge, in this paper, we review the notions of connectivity, intrinsic computation, criticality and coherence—the 4C's of the brain at rest—from a complexity science perspective.

## 2. Background

### 2.1. Complexity in the brain

Conceptualizing the brain as a complex system is an especially powerful way to investigate the spontaneous ongoing dynamics of the brain [17]. While the exact definition of a complex system is still under debate [17,18], the brain satisfies the four properties all systems characterized as ‘complex’ necessarily share [19]:

1. Multiplicity and interdependence: the brain is made of small subunits that interact with each other through a vast network of local and long-range connections.
2. Nonlinearity: the interactions between neural elements are often nonlinear, giving rise to rich dynamical phenomena.
3. Self-organization: the activity of the multiple brain subunits develops into structured patterns spontaneously, in the absence of any form of centralized control mechanisms.
4. Emergence: the macroscopic behaviour of coordinated brain activity cannot be understood purely in terms of the neuron-to-neuron interactions.

This perspective allows us to bring the sophisticated conceptual machinery of complexity science to the study of the brain, while extending the repertoire of techniques employed in neuroimaging analysis with tools specifically designed to fully exploit the richness of such datasets.

At a high level, we will consider two distinct approaches to brain complexity: one from the perspective of nonlinear DST, and one from the application of IT. In the former, complexity is associated with dynamical instabilities giving rise to pattern formation, self-organization and metastability [20–22]. In the latter, complexity is related to the statistical structure of brain activity, typically quantified with tools derived from entropy or mutual information (MI) [23]. In order to situate our 4C's within these approaches, we provide in the next sections a brief overview of the intuitions and terminologies native to these two approaches.

### 2.2. Dynamical systems theory

**Figure 1.**
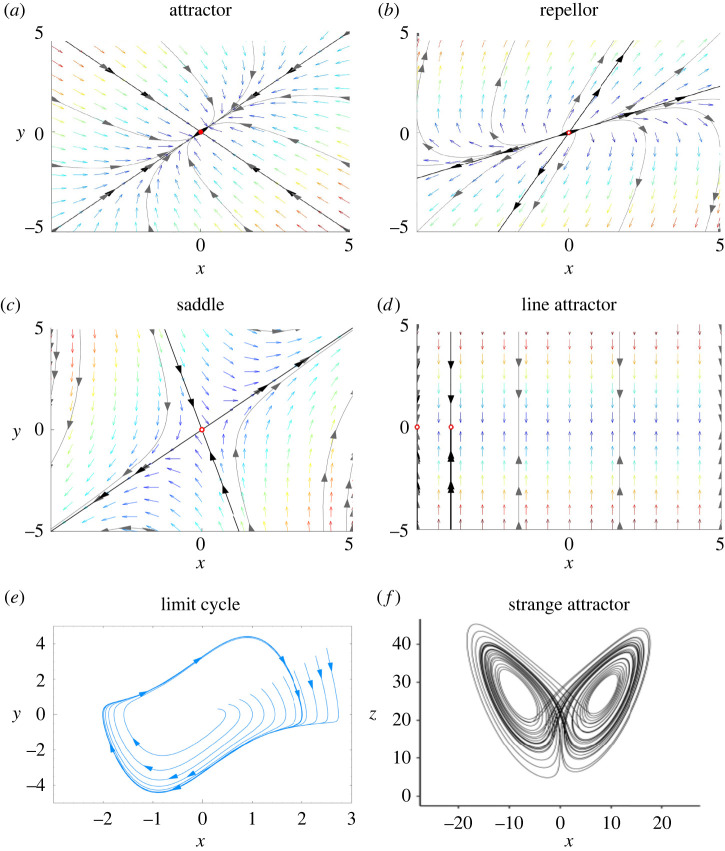
Phase portraits—geometrical representations of trajectories in dynamical systems with different attractor types (with or without vector fields). A phase portrait is a plot of the trajectories of a dynamical system. Colour-coded slope or vector fields represent the rate of change of the trajectory at that point with respect to time—deep red indicates high rates of change and dark blue indicates low rates of change. Examples of trajectories for different initial conditions are shown in black. (*a–d*) Produced with Phase Portrait Plotter [26]. (*e*) XaosBits at English Wikipedia, CC BY 2.5, https://commons.wikimedia.org/w/index.php?curid=732841. (*f*) The Lorenz attractor is an example of a strange attractor [27].

A dynamical system is a system that changes over time in a way that can be described by a single or a set of differential equations [24]. If there are nonlinear interactions among the variables of the system, the system is described by nonlinear equations and is referred to as a *nonlinear dynamical system*. Given a set of initial conditions, the solution to the differential equation(s) can be plotted in a phase diagram that illustrates the temporal evolution or trajectory of the system. These trajectories exist within an *n*-dimensional *phase space*,^1^ which gives an account of the possible solutions that the system could potentially adopt—with *n* reflecting the dimensionality of the representation of the system. If a family of trajectories (i.e. solutions of the equations starting from different initial conditions) flows towards a particular region of phase space, that region is known as an *attractor*. If small perturbations in the system eventually return the system to its current attractor, the attractor (solution) is said to be stable. Conversely, if the trajectories flow away from a region, that region is considered a repellor. If trajectories flow towards an attractor in one dimension, but away from it in another, the region is referred to as a saddle point. Attractors may be fixed points, fixed lines, stable or unstable limit cycles, or when characterized by more complex forms, they are termed strange attractors [24] as illustrated in figure 1.

Thinking in terms of attractors enables some useful taxonomies for dynamical systems: a *multi-stable* system is a system with more than one attractor; a *metastable* system is a system with a saddle which may be linked in sequence with other saddles; and finally, a *critical system* is a system that has an attractor that responds to small perturbations with long and unstructured excursions [28] (the notion of criticality is developed in a later section).

A system exhibits *dynamic stability* if it reliably returns to its steady-state attractor after perturbations. A parameter of the system that is capable of driving the system out of dynamic stability is called a *control parameter*. At a point of instability, a *bifurcation* often occurs—where a single attractor divides in two or changes shape, and solutions that were previously stable become unstable.

The preceding description adheres to the terminology of mathematics. In statistical physics, a variable in a dynamical system is known as a state variable; and a bifurcation is referred to as a *phase transition* [29]*.* Water turning into ice at 0°C is an example of a first-order phase transition where the density of H_2_O changes abruptly at the freezing point (resulting in the well-known increase in volume). By contrast, an iron magnet changing from being ferromagnetic to paramagnetic at 770^o^C is an example of a second-order phase transition. The net magnetization does not change abruptly at the Curie temperature; rather, at the critical point there is a divergence of the magnetic susceptibility, resulting in large fluctuations of the magnetization. Hence, first-order phase transitions display a discontinuity on an observable (e.g. density), while second-order transitions display discontinuities in some of their derivatives (e.g. the rate of change of the magnetization with respect to an external field) [30].

### 2.3. Information theory

In the dynamical systems framework, relationships between variables are typically described in terms of differential equations. A complementary description investigates the interactions between the variables through their statistical interdependencies. These interdependencies may be probed via statistics and probability theory, and more specifically through the framework of *information dynamics* [31,32], which aims to describe complex dynamical interdependencies via MI [33,34] and related tools.

In general, the (statistical) entropy of a signal measures its degree of variability or diversity. Similarly, the MI between two signals captures their covariation—or more precisely, to what extent the observed values are different from the values from similar but statistically independent signals. Using these basic building blocks, a range of different extended measures of interdependencies can be built. For example, transfer entropy corresponds to how well the future of a ‘target’ signal can be predicted from the past of a ‘source’ signal, over and above how well the target signal predicts itself [35,36]. As another example, there exist various multivariate extensions of the MI that capture high-order (i.e. beyond pairwise) interactions, which can be characterized as assessing different types of covariation [37], or via other mathematical principles such as cohomology [38].

An important feature of information-theoretic tools is their great flexibility: these tools can be applied to ordinal, categorical, and continuous data from linear and nonlinear systems. Their generality and wide range of applicability turns the notion of information into a powerful ‘common currency’, through which interdependencies in different systems can be characterized and compared [31,37,39,40].

It is important to bear in mind that, in these types of applications, information-theoretic quantities are not conceptualized in terms of optimal solutions to engineering problems of data transmission (i.e. Shannon's characterization), but as descriptions of states of incomplete knowledge—following the seminal work of Jaynes [41]. Therefore, measures like statistical entropy, MI, transfer entropy and multivariate information measures are understood not as actual bits being transferred through a communication channel [42], but as inferential statements about the statistical structure of the system of interest [43].

## 3. The 4C's: conceptual approaches to spontaneous brain dynamics

Building on a view centred on complexity, dynamical systems and IT, the following sections review various approaches to analyse brain activity captured with fMRI at rest from a complexity-science perspective. Furthermore, we discuss recent findings where this approach revealed novel relationships between DST and information dynamics.

### 3.1. Connectivity: networks and patterns

Experimental evidence accumulated in recent years has revealed that variation in structure and function in the human brain (both for normal development and for disease) tends to be widely distributed, and hence there is often no simple one-to-one mapping between cognitive functions and individual brain regions [44–47]. An important paradigm shift in neuroimaging research was therefore to go beyond mere activation and focus instead on *co-activation,* i.e. on the interdependency between the activity of multiple regions. The fact that the latter approach is providing rich empirical results is likely related to the anatomical configuration of the central nervous system, which constitutes an organized network of axonal tracts between distinct grey matter regions. This evolving map of structural connections between neuronal elements is known as the (structural) ‘connectome’ [48,49], which can be assessed non-invasively *in vivo* via probabilistic or deterministic tractography based on diffusion-weighted MRI imaging [48].

Contrasting with structural connectivity (SC), which refers to the physical anatomical connections between regions, functional connectivity (FC) refers to the statistical relationship of coordinated activity in spatially distant regions [50]. Before moving on to more elaborate (and perhaps more principled) approaches to connectivity, we will briefly review the origins of FC. Temporal correlations of slow spontaneous fMRI fluctuations of less than 0.1 Hz between brain regions were initially observed in the early 1990s during task execution [51]. Similar correlations were then observed between the left and right sensorimotor cortices when the brain was at rest [52]. Since then, it has been shown—initially through positron emission tomography, and now more commonly with fMRI—that spontaneous activity reflects sustained functionality in the form of a ‘default mode’ of intrinsic connectivity [53]. Patterns of resting-state temporal correlations have been extracted and documented as resting-state networks (RSNs) [53–55] using a multitude of methods.

Evolving from FC, whether static or time-varying [56], a number of different forms of connectivity have been defined and named to distinguish them from canonical statistical relationships. Effective connectivity, evaluated, for example, via dynamic causal modelling [57,58], aims to infer the causal architecture of dynamical systems. Time-varying or dynamical FC (dFC) has also progressed beyond statistical relationships in temporal correlations. Indeed, dFC has been elucidated with phase relationships [59–61] and information-theoretic measures [62], which are in principle more amenable for investigations in nonlinear dynamical systems [63,64].

**Figure 2.**
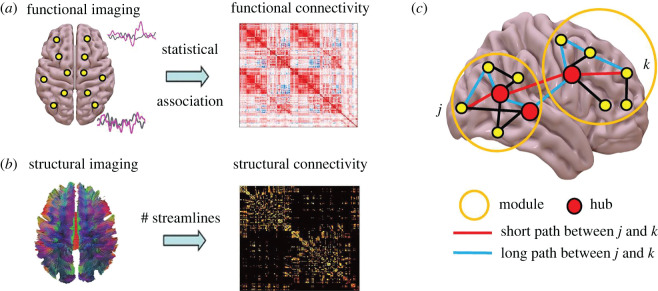
Connectivity in the human brain. (*a*) Functional connectivity can be quantified from functional MRI, as the linear correlation statistical relationships between BOLD signals. (*b*) Structural connectivity can be quantified from diffusion-weighted MRI, as the white matter streamlines between regions. (*c*) Graph theory can characterize local properties of individual nodes (e.g. high-degree nodes or ‘hubs’) but also mesoscopic properties (e.g. modular organization) and macroscale (e.g. average shortest path length in the network).

Although the word ‘network’ is usually used to define resting-state FC, the tools of graph theory (such as community structure [65]) can be applied to a range of scenarios. For example, from a complexity science perspective both SC and FC (however defined) can be conceptualized as networks—considering systems of neuronal ensembles or regions to be nodes (or vertices), connected by links (edges) corresponding to either axonal projections (SC) or FC relationships [66,67] (figure 2). As a matter of fact, the broad application of graph theory to a range of aspects of brain imaging data has given birth to the emerging field of network neuroscience [68–71].

Studies following this approach have shown that healthy brain SC and FC networks exhibit a host of complex network features, including small-worldness [72,73], short path lengths, high clustering, hubs [66], a rich club [74], a diverse club [75], within a modular and fractal (self-similar) community structure [76–78]. Examples of graph-theoretic metrics that are commonly employed in neuroscience may be found in electronic supplementary material, table S1. The ‘rich club’ of strongly interconnected bi-hemispheric hub regions includes superior parietal and frontal cortices, precuneus, putamen, hippocampus and thalamus, which are considered to be crucial for whole-brain communication [74].

Overall, conceptualizing the brain in terms of structural and functional networks has made it possible to import into neuroscience a number of well-developed mathematical tools from complexity science, providing a much-needed framework to study the brain as the interconnected system it is.

### 3.2. Computation and information dynamics

#### 3.2.1. Integrated information and partial information decomposition

The great flexibility and interesting interpretability of information-theoretic measures have triggered a large range of investigations of the human brain using such tools. Early efforts in information-theoretic analysis of resting brain activity endeavoured to formalize the notion of ‘dynamical complexity’, understood as the simultaneous occurrence of functional segregation and global integration in the brain [79]. Systems exhibiting both high integration and a high specialization exhibit functional patterns of the highest dynamical complexity. Subsequent work inspired by further developments of these ideas led to a measure of ‘integrated information’, *Φ*, that quantifies the ability of a system to carry information as a whole beyond what is carried by its parts [80], which leads towards novel and practical measures for data analysis [81,82].

However, the intuition that information can only be transferred or stored between parts of a system fails to capture the full range of possible information dynamics, as noted, for example, by James *et al.* [42]. Another important extension of classic information-theoretic tools to capture such higher-order interactions is provided by the framework of partial information decomposition (PID) [83].^2^ PID proposes a formal decomposition of the MI provided by various predictors about a target variable, introducing three fundamentally distinct types of information: redundant, unique and synergistic. Intuitively, unique information refers to information that is provided by one predictor but not the others, redundancy refers to the case where multiple predictors each provide the same information about the target, and synergy refers to predictive information that only becomes available if the predictors are considered together. There is an ongoing discussion on how to best calculate these different types of information in practice, which constitutes an active area of ongoing research. However, despite their technical differences, many of these proposals do not differ much in practical setups [84,85], and have been proven successful in various applications.

This powerful extension of classic IT has already found successful application in neuroscience; for example, PID has been used to assess synergistic information processing in an organotypic culture of spiking neurons [86], to link information storage and information transfer to sub- and supercritical regions of phase transition in a neuronal population oscillator model [87], and to show that rich club neurons perform 160% more computation than non-rich club neurons [88]—providing avenues to combine the information-dynamic and graph-theoretic views of the brain.

#### 3.2.2. Applications of *Φ*ID: integrated information decomposition

However, the framework of information dynamics introduced by PID is restricted to scenarios with a single target variable, being unable to discriminate between different ways in which two or more target variables can be affected collectively. To account for how multiple variables jointly affect one another's temporal evolution, a further generalization is needed. Building on intuitions from both *Φ* and PID, the framework of *integrated information decomposition* (*Φ*ID) proposes an encompassing taxonomy for the diverse information dynamics phenomena that can take place in complex stochastic systems [82], [Preprint] [89]. *Φ*ID has two main features: it can be used to decompose and hence better understand existent measures of complexity; and it can provide the fundamental building blocks to tailor new measures that track specific processes of interest. Such new measures have been able to reconcile the dynamical systems and information dynamic views of complexity. As an example of the former, *Φ*ID has been used to show that existing quantifications of integrated information (and related quantities such as ‘causal density’ [90]) are not capturing a unique type of information dynamics, but rather conflating multiple ones. Following this finding, a revised version of *Φ*—denoted by *Φ*^R^—has been proposed, which has been shown to capture various important aspects of a broad range of complex systems [91,92], and be more precise than *Φ* in finding or detecting specific differences between conscious and unconscious dynamics of the human brain [93].

More broadly, *Φ*ID is enabling novel ways to conceptualize brain function, by providing an *information-resolved* FC that complements the *time-resolved* perspective [93]. Empirical analyses based on fMRI data have shown a role for redundancy in ensuring robust input/output communication channels, as is especially prominent in somatomotor and sensory regions. By contrast, synergy is related to efficient communication in high-order association cortices, and supports humans' sophisticated cognitive functions, being more prominent in evolutionarily expanded regions of the cerebral cortex [93]. The further identification of specific molecular, cytoarchitectonic and metabolic profiles suggests that in the close future we may be able to embody information processing properties into tissues’ biophysical properties, a critical step towards supporting the biological plausibility of intrinsic computation in the brain.

Crucially, *Φ*ID also provides a mathematical framework as a basis for formalizing *causal emergence*. This framework has shown the emergence of motor information from electrocorticography recorded from a macaque's motor cortex, and also that emergent dynamics are more prevalent in healthy controls rather than in subjects with serious brain lesions. Therefore, *Φ*ID provides a way to address two of the key aspects of a complex system outlined in [19] and [17]: namely, multiplicity and emergence.

### 3.3. Criticality

#### 3.3.1. Origins and meanings

Criticality refers to scenarios where collective properties of a system composed of many parts exhibit an abrupt change—akin to the freezing of water when temperature goes below 0°C. In general, a system is said to undergo a phase transition when a small change in a control parameter (e.g. temperature) causes a large collective change (e.g. freezing). In textbooks, criticality is often illustrated through canonical examples, most prominently the Ising spin model [94], which possesses a well-studied phase transition between disordered and magnetized states. Importantly, besides the abrupt change in the order parameter that defines them, a system that is near a phase transition (or *critical point*) displays a host of unusual features: long-range correlations, power law (also known as *scale-free*^3^) statistics, fractal structure, long transient periods and sensitivity to external perturbations—to name a few [28,30,95]. Furthermore, theoretical and computational work has demonstrated that systems poised near criticality can exhibit several advantageous computational properties, such as an extended range of responses to inputs [96] or increased transfer of information [97]. It is for these reasons that some have suggested the idea of criticality as a universal guiding principle for brain organization [30,98,99].

**Figure 3.**
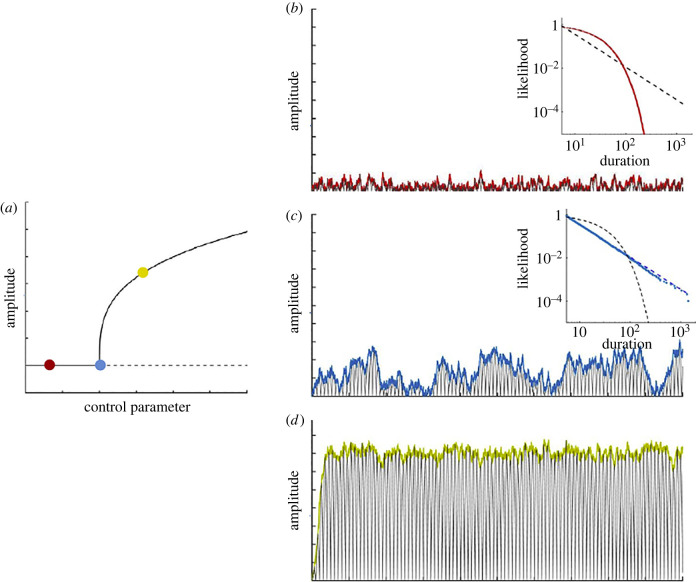
Criticality in a low-dimensional system consisting of a few interacting components. (*a*) Diagram of a supercritical bifurcation depicting the amplitude of a system's state variable (*y*-axis) as a function of a control parameter (such as the strength of interactions, *x*-axis). When the control parameter is increased, the activity of the system switches from subcritical (red circle) to a supercritical regime (yellow circle). The point of change is known as the critical point (blue circle). (*b*) In the presence of noise, the subcritical system (red circle) systematically returns to the steady state equilibrium with rapidly decaying amplitude. The duration of high amplitude events follows an exponential probability distribution (red dots, inset). (*c*) At the critical point, the amplitude fluctuations have high variance, rising and falling slowly due to the transient stability of oscillations. The relationship between the duration and the likelihood of high amplitude oscillations follows a power law distribution, corresponding to a linear relationship in double logarithmic coordinates with a slope described by a critical exponent of −3/2 (inset). (*d*) Beyond the critical point, the system is in a supercritical regime and exhibits sustained oscillations with small amplitude variability. Adapted from [28] with permission from Elsevier.

One important distinction is that between *statistical criticality*, in which a system is in a critical state and follows the statistical patterns discussed above, and *dynamical criticality,* in which the system is close to a phase transition, or in mathematical terms, lies close to a dynamical bifurcation, where small deviations can lead to an abrupt change in its dynamical behaviour. The position of a system with respect to the bifurcation defines whether it is classified as *subcritical* (the system has a steady state equilibrium but responds to perturbation with dampened oscillations) and *supercritical* (where the stability of the steady state equilibrium is lost and oscillations are self-sustained). At the bifurcation point, the equivalent stability of both steady-state and oscillatory equilibria drives large amplitude fluctuations exhibiting statistical patterns of critical systems (figure 3).

The idea that critical states are particularly important in nature received further support with the idea of self-organized criticality, which postulates that dynamical systems can self-organize into a critical state spontaneously, without careful fine-tuning of a control parameter [100]. Unfortunately, applying these principles in practical scenarios is a challenging endeavour. In fact, it is known that some systems may display some, but not all, of the properties of the classical critical models; and that exotic phenomena can take place in systems with heterogeneous components, like smeared phase transitions [101] or Griffiths phases [102]. Furthermore, while statistical and dynamical criticality are often related, they can also occur independently of each other [103]. It is for these reasons, among others, that the study of criticality in neural systems is rife with difficulties and misunderstandings.

#### 3.3.2. Criticality in the brain

Despite significant challenges, over the last 20 years scientists have found evidence for multiple signatures of criticality in neural data from multiple modalities. Arguably, the first evidence of this kind was found in human brain oscillations in the large variability, long-range temporal correlations, and power law scaling of electroencephalography (EEG) amplitude fluctuations in the 10–20 Hz frequency range [104]. Mesoscale signatures of criticality were demonstrated in the scale-free power law distribution of size and life-time of neuronal avalanches in mature organotypic cultures and acute slices of rat cortex [105]. These local synchronized activity patterns were confirmed to be scale-free in awake non-human primates [106]; this finding was an important intermediate transition towards demonstration of avalanches in human neuroimaging. Subsequently, scale-free avalanche dynamics were found in resting magnetoencephalography (MEG) [107].

More recently, scale-free avalanche dynamics were found in a combined broadband analysis of MEG and EEG, confirming that broadband resting state activity measured with MEG and EEG can be described as a series of neuronal avalanches [108]. At a larger scale, studies in resting-state fMRI have shown avalanches of activated clusters of voxels using an innovative point-process [109]. Scale-free power law distributions have also been found in the life-time of phase-locked activity between different brain regions in fMRI and MEG [110], although these findings have been contested [109,111]. Returning to long-range temporal correlations, these amplitude correlations in the 10–20 Hz frequency range have been found to correlate with behavioural scaling [112], and to reproduce in broadband signals [108]. In addition to temporal correlations, long-range spatial correlations were found in resting state fMRI using spatial coarse graining [113].

**Figure 4.**
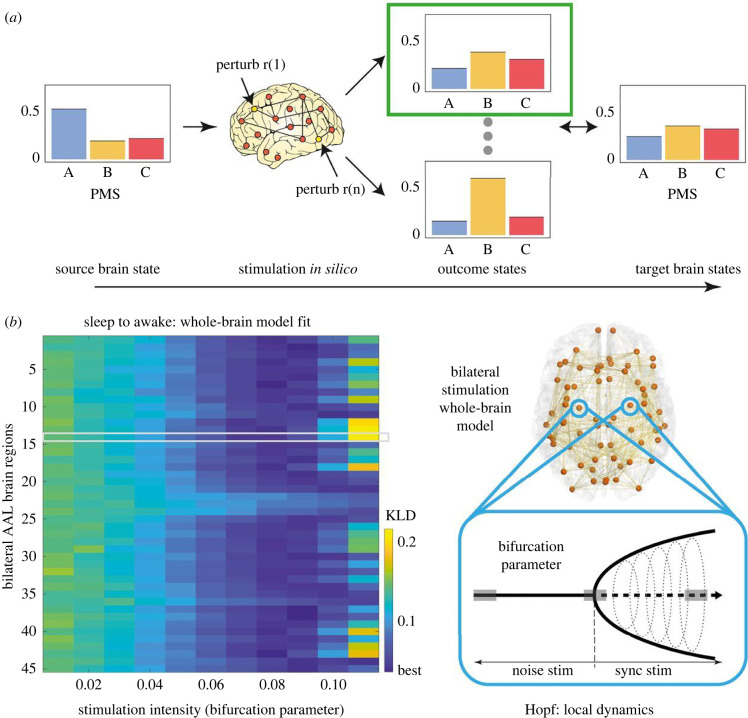
Schematic of strategy for forcing transition between source and target brain states. (*a*) The brain regions in the whole-brain model of the source state can be systematically stimulated, and the results can be compared to the target state. Specifically, in the local-region Hopf model, it is easy to perturb the model by simply changing the bifurcation parameter [121]. (*b*) The stimulation intensity, i.e. the strength of the perturbation, is directly related to the amount of shifting of the local bifurcation parameter [123]. The composite results are shown left of stimulating the whole-brain EC model bilaterally. The KL distance obtained for brain state transition fitting when perturbing separately each of the 45 AAL regions (using bilateral stimulation) with different stimulation intensities in source state (deep sleep) is shown. Here, one region is highlighted in a grey region, which is being stimulated while the other regions are kept at their normal bifurcation parameter. The colour scale for the results shows the level of fitting with the target state (wakefulness), i.e. lower values (deep blue) correspond to the most effective transitions. Hopf model—a computational model that emulates the transition from noisy to oscillatory dynamics as the bifurcation parameter changes; KL, Kullback–Leibler; AAL, automated anatomical labelling [124]. Adapted with permission from [122].

Interestingly, these signatures of criticality seem to be characteristic of healthy, conscious brain dynamics. Deviations from critical dynamics were found in MEG in interictal epileptiform activity in epilepsy patients [114] and disruption of long range temporal correlations has been found in patients with major depressive disorder [115], early-stage Alzheimer's disease [116] and in healthy adults during deep sleep [117]. During loss of consciousness, the long-range amplitude correlations across brain regions disappear [118] and overall the brain appears to move away from dynamical criticality towards greater dynamic stability [119]. By contrast, during consciousness dynamical criticality is maintained as the underlying dynamical modes hover in the vicinity of the critical stability threshold [120]. These findings are consistent with a computational model of the difference in stability between awake and sleep states [121]. The dynamic stability in the sleep state was reflected in a rigidity to external perturbations. By contrast, the awake state was reflected in a longer integration of the perturbations and a slower return to equilibrium dynamics. Building on these findings, a further study showed that shifting certain dynamical modes in the model towards their critical stability threshold through local bifurcation parameter changes, stimulated switching between sleep and awake states [122] as shown in figure 4. Although the studies of Solovey *et al.* [119] and Deco *et al.* [121,122] were not related, the results demonstrate the linkages between critical control parameters at the level of individual regions or dynamical modes, and the resulting whole-brain dynamical criticality or stability.

Considering the previous discussion on information dynamics, it is interesting to note the link between neuronal avalanches and computation. In neuronal cultures and non-human primates, a special type of neuronal avalanche called *coherence potential* was identified when a threshold function was applied to binarize local field potentials [125]. *Coherence* in this sense refers to the resulting waveform (similarity of local field potentials), and *potential* reflects their all-or-none behaviour, characteristic of action potentials. These coherence potentials propagate macroscopically keeping their amplitude and waveform, involving both short- and long-range cortical co-activations [125]. The emergence of coherence potentials has been likened to the emergence of gliders in Conway's Game of Life [126], and theorized to play a role in information transfer within the cortex at the network level [127,128]. This hypothesis alludes to a powerful link between criticality and computation, which we believe deserves more investigation.

Nonetheless, the concept of criticality in the brain is not without controversy [129]. This stems from its occasional metaphorical use without robust support of empirical data [98,130], and fundamental challenges to power law statistics and universal scaling as critical phenomena [131]. Moreover, recent proposals suggest that the brain may not be in a critical regime, but rather in a quasi-critical [132] or a slightly subcritical regime [133]. Overall, it is clear that while these difficulties continue to generate heated debate, the theory of dynamical criticality and phase transitions brings extremely useful tools to neuroimaging, which can be used pragmatically to characterize brain dynamics across states.

### 3.4. Coherence and synchronization in the brain

The concept of coherence is fundamental for investigating fluctuating quantities in many scientific disciplines [134]. Although the term is commonly used in neuroimaging research, the precise meaning and mathematical definition varies across studies. For example, FC has been estimated with *time–frequency coherence* derived from signal magnitude and phase as a function of time and frequency [60], and with *phase coherence* derived from instantaneous phase differences as a function of time [123]. For narrow band-pass filtered signals, *coherence* has been defined as the correlation coefficient between two signals [134], and *phase synchrony* as the instantaneous phase difference between two signals [135]. Using this definition, Varela proposed that dynamic connections, mediated by synchrony between distributed networks, create transient neuronal assemblies that facilitate large-scale integration of functionally specialized brain regions [135].

To understand synchrony, one must first recognize that neural activity is mainly oscillatory due to nonlinear interactions between neurons, with the resulting aggregation of electrical signals producing rhythmic activity at distinct frequencies. Such activity has been measured in spiking neurons, local field potentials and macroscopically with EEG, MEG and fMRI [1]. Oscillations in the gamma frequency (30–60 Hz) reflect the collective firing of action potentials in pyramidal excitatory neurons, coordinated via the delayed negative feedback of gamma aminobutyric acid (GABA) interneurons [136]. This coupling of pyramidal and GABA neurons is known as the pyramidal interneuronal network gamma (PING) network. GABA interneurons also link PING networks into and across individual cortical columns, creating a mechanism for the spread of rapid synchronization and desynchronization across the cortex [137]. It has been shown in computational and empirical studies that these gamma rhythms generate the slow macroscopic whole-brain dynamics observed in MEG and fMRI [138–140]. Varela proposed that canonical motifs such as the PING network were matched by parallel phase synchrony [135].

**Figure 5.**
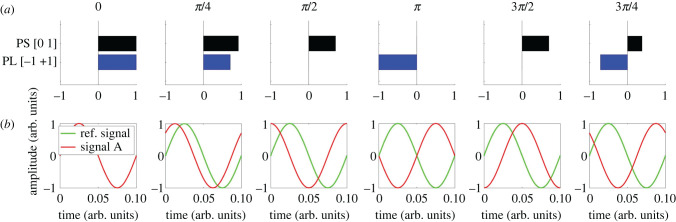
The phase relationships of phase synchrony and phase-locking. Phase synchrony (PS) reflects the degree of synchronization between the two signals, while phase-locking (PL), evaluated as the cosine of the relative phase, reflects the degree of phase alignment and is sensitive to both in-phase and anti-phase relationships between the two signals. (*a*) Values of PS and PL for different phase shifts between a reference signal and signal A. (*b*) Visualization of the reference signal and signal A waveforms over time for different phase shifts.

To avoid confusion with the terms of coherence, phase coherence and phase synchrony, we prefer to discuss the different *phase relationships* that may exist between distributed neuronal assemblies, or in the context of whole-brain studies, between distributed brain regions. We will focus on two common and complementary measures of phase relationships (figure 5).

#### 3.4.1. Phase synchrony and the Kuramoto order parameter

The Kuramoto model of coupled oscillators is a classic model of mathematical physics, being a simple model that is capable of displaying phase transitions and other interesting collective phenomena of great relevance for the study of brain dynamics [141]. The *Kuramoto order parameter* (KOP) is a metric of instantaneous phase synchrony that encapsulates the collective behaviour of the group of oscillators. Additionally, the time average of this order parameter is a measure of global synchrony, and the variability of the order parameter over time provides a proxy measure of metastability (discussed in detail in the next section). Phase synchrony refers to phase uniformity between neuronal assemblies, being calculated as the magnitude of the average phase. As such, phase synchrony of 1 represents full synchrony while 0 corresponds to complete asynchrony. The KOP has been used to compare global synchrony and metastability in a pharmacological challenge [142], to predict clinical symptoms in schizophrenia [143], and to assess the emergence of cluster synchronization in semi-empirical computational models of coupled oscillators [139].

#### 3.4.2. Phase-locking

Phase-locking refers to a constant relationship between the phases of two neuronal assemblies, commonly calculated as the cosine of the phase difference between two signals. Hence, phase-locking of −1 and +1 represents anti-phase-locking and in-phase-locking, respectively. Phase-locking has been used to identify community structure in fMRI resting state data through dynamic FC [59,144]. Neuronal coherence, measured with phase-locking, underlies the hypothesis that distant neuronal communities do not necessarily need to be synchronized (phase synchrony), since (phase-locked) coherence is sufficient for optimal transfer of spiking-encoded information between the sender and the receiver. Although this communication-through-coherence (CTC) [145–147] hypothesis was proposed for gamma-mediated microscopic neuronal assemblies, the signature of an underlying gamma-band coherence mechanism has been observed in semi-empirical fMRI data [148].

The complementary phase relationships of phase synchrony and phase-locking have recently been investigated in resting state fMRI data. Using a complexity-science approach, a battery of metrics derived from theories of dynamical systems, stochastic processes and information dynamics was developed to characterize resting state dynamics. Novel relationships between the metrics were revealed, allowing a predictive model for *Φ*^R^ to be constructed using metrics from dynamical systems and IT. Overall, the study showed the complementary utility of phase synchrony and phase-locking, and revealed that the majority of fMRI resting-state characteristics reflected an interrelated dynamical and informational complexity profile [92].

## 4. Discussion

Viewing the brain as a complex system complements alternative domain-specific models of spontaneous brain activity. With a focus on mesoscale interactions leading to macroscale phenomena, complexity science provides a rich set of concepts, theories and methods from disparate scientific disciplines, which can be used for investigative and explanatory studies of brain dynamics. Complexity in the brain has been associated with dynamic instabilities that give rise to pattern formation and self-organization [22] and with balancing the dialectic dynamics of regional functional segregation with global coherent integration [79]. Taking this into consideration, we have reviewed the key concepts of DST, network science and information dynamics, and illustrated how these notions provide complementary perspectives on the temporal evolution, organization and interactions of macroscopic brain components. We have shown how network science expands on the limitations of canonical FC through identification and quantification of network properties. The notion of criticality was addressed from the perspectives of statistical and dynamical criticality, and the role of perturbation analysis to investigate criticality in both empirical and semi-empirical computational models was discussed [149]. Computation in the absence of tasks, intrinsic computation, was reviewed and shown to be characterized in terms of synergistic and transfer information flows, and quantified by *Φ*^R^. Coherence was discussed under its many guises and the complementary nature of phase synchrony and phase-locking was explored. Finally, metastability was introduced as the standard deviation of the Kuramoto order parameter.

Metastability appears to be universal across the 4C's of complex brain dynamics, although it is not yet clear if metastability plays an enabling or a defining role for healthy brain functioning. Metastability expresses a healthy tension between the competition for functional specialization and global coordination in the brain [150]. Indeed, when we review our 4C's we see that for *connectivity*, metastability was maximized for a community and small-world structure [151], reduced after damage to SC following traumatic brain injury [152], and when at a maximum, revealed a dynamic core in FC [123]. For *criticality*, statistical indicators of criticality have been observed when metastability was at a maximum [109,110,153]. For *computation*, global synchronization and *Φ*^R^ were found to be intimately dependent on metastability [91,92]. Importantly, cluster synchronization, also dependent on metastability, has been shown to drive the transient emergence of collective oscillations, replicating features of resting-state MEG [139]. For *coherence*, CTC-like routes of communication were found to be optimized when metastability was at a maximum [148].

**Figure 6.**
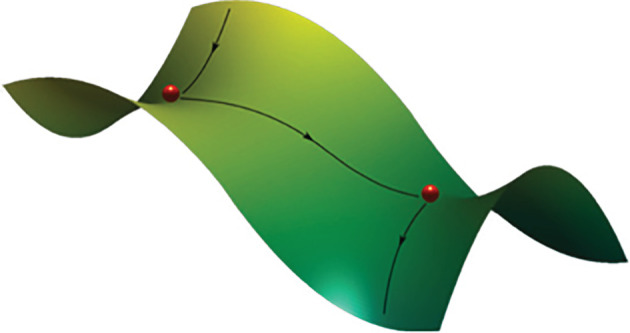
Representation of a path that joins metastable states. In the phase space of a dynamical model, a metastable state is represented by a saddle fixed point. Based on this landscape metaphor, it is easy to see that two saddles can be connected by an unstable one-dimensional saddle. Adapted from [155] with permission from Elsevier.

Metastability has been described as a subtle blend of segregation and integration among brain regions that show tendencies to diverge and function independently, as well as tendencies to converge and function collectively [150]. In dynamical systems, these tendencies occur when a system moves away from a stable equilibrium or attractor and exhibits transient dynamics while remaining away from any attractor of the system [154]. Trajectories in the metastable regime follow a sequence of metastable states or saddle nodes (see §2.2) on a path that joins different equilibrium points, where the unstable dimension of one saddle node is the stable dimension for the following saddle node as illustrated in figure 6 [156]. The system moves between saddle nodes without the need for energy consuming disengagement mechanisms [157], and so freely explores a repertoire of metastable states until changes in the control parameter move the system to a different regime of mono- or multi-stability [157].

This highly theoretical conceptualization of metastability has not prevented its investigation in computational and semi-empirical models, and in empirical imaging data. A first quantification of metastability goes back to the late 1990s where it was estimated as the entropy of the spectral density of a time series [158]. However, it was the appearance of the seminal article on the Kuramoto model [141] that led to the now most common quantification of a proxy for metastability as the standard deviation of the KOP^4^ [138,151,159,160]. Computational models have used this metric of metastability to investigate the relationship between the amplitude modulation envelopes of MEG and the slow fMRI signal [139], to study the effect of lesions on functional brain dynamics [161], to reveal the coincidence of metastability with integrated information [91], and to show that the brain at rest operates at maximum metastability [123]. Furthermore, CTC-like routes of communication, as discussed in §3.4, emerged when metastability was at a maximum in a semi-empirical model of whole-brain dynamic FC [148], putting forward a possible mechanism for flexible communication within a fixed SC.

Studies of metastability have not been confined to computational models. In empirical studies, metastability was shown to be at a maximum when the brain was at rest [162], being reduced during states of unconsciousness [163], and increasing beyond the resting state value during psychedelic states [142,164]. Metastability has also been shown to predict clinical symptoms of schizophrenia [143], reduce progressively for mild cognitive impairment and Alzheimer's disease [165], and be correlated with cognitive flexibility [152] and high-order cognitive ability [166].

In summary, metastability is ubiquitous across diverse models of brain functioning in resting state. Proxy measures of metastability and *Φ*^R^ provide complementary quantification of complexity, and reveal novel relationships between dynamical and informational complexity [91,92]. It may now be time to better understand the physics behind metastability [167] and so improve the proxies used to measure this universal phenomenon in resting state brain dynamics.

## 5. Conclusion

Unravelling the mysteries of coordination and communication in RSNs has attracted attention in the scientific disciplines of mathematics, statistical and theoretical physics, network science and IT. This classical leakage of discipline-related methods into neuroimaging research is welcome, despite the resulting introduction of a plethora of terminology and concepts which are not always applied in their strict scientific sense. Under the auspices of complexity science, this review has attempted to disentangle the ‘4C's’ most commonly found in resting-state fMRI literature, locate them in their respective disciplines and conceptualizations, and highlight their complementarities and intersections. The nebulous concept of metastability was shown to be universal across these 4C's and symbiotic with *Φ*^R^ as a proxy for complexity. Accepting the plausibility and legitimacy of different models of brain functioning, and embracing their eclectic methods and tools, should ultimately lead to improved descriptions, and eventually to understanding and prediction for healthy and disordered brain functioning.

## Data Availability

This article has no additional data.

## Footnotes

1 For historical reasons, the term ‘phase’ here does not refer to the phase of an oscillation, but more in general to the possible phases that any system can explore [25].

2 The pioneering work from Williams & Beer [83] initiated a subfield of information theory and has been cited in more than 300 peer-reviewed publications.

3 Scale-free denotes that a system does not possess a characteristic time or length scale. Statistically, the system exhibits a power law probability distribution where the constant exponent *k* in *y* ∝ *x*^−*k*^, the critical exponent, is less than 2.

4 The reasoning behind this proxy is that both extreme values of the KOP (0 and 1) correspond to equilibrium points of the model (desynchronized and hyper-synchronized, respectively). Thus, the standard deviation of the order parameter quantifies how widely it fluctuates between the vicinity of these two equilibrium states.
